# Maternal social support and child developmental outcomes: an analysis of the Born in Bradford cohort

**DOI:** 10.1136/archdischild-2025-328885

**Published:** 2025-06-24

**Authors:** Lucy Barrass, Laura D Howe, Sunil S Bhopal, Josie Dickerson, Rachael W Cheung, Tom D Allport

**Affiliations:** 1 Population Health Sciences, University of Bristol, Bristol, UK; 2 Born in Bradford, Bradford Institute for Health Research, Bradford, UK; 3 Department of Population Health, London School of Hygiene & Tropical Medicine, London, UK; 4 Centre for Academic Child Health, University of Bristol, Bristol, UK; 5 Community Children’s Health Partnership, Sirona CIC, Bristol, UK

**Keywords:** mental health, child development, child health, epidemiology

## Abstract

**ABSTRACT:**

**Background:**

Women lacking social support during pregnancy often have worse mental health, but we know little about the influence of social support on child development, or the impact for migrant women. We aimed to investigate the association between maternal social support during pregnancy and child development using the Born in Bradford birth cohort.

**Methods:**

Social support was evaluated using a composite score of items from the baseline pregnancy questionnaire. Outcomes were Communication and Language and Personal, Social and Emotional development school-readiness assessments from the Early Years Foundation Stage Profile (EYFSP, age 4–5 years) and the Strengths and Difficulties Questionnaire (SDQ, age 7–11 years), with associations tested using logistic and linear regression models. We explored the modifying effect of maternal migrant status.

**Results:**

3257 and 1413 cohort participants had EYFSP and SDQ outcomes, respectively. Higher levels of social support were associated with better EYFSP outcomes and SDQ scores. One SD higher social support score was associated with 13% lower odds of missing any EYFSP communication and language target (95% CI 0.79 to 0.95); 17% lower for EYFSP personal, social and emotional development (95% CI 0.75 to 0.92) and 0.65 (95% CI −0.98 to –0.32) lower overall SDQ scores, after adjustment for all variables. There was some evidence that maternal migrant status modified associations with SDQ, but not with EYFSP outcomes.

**Conclusions:**

Greater attention to the role of social support in pregnancy, and its social and cultural context, may be helpful in developing and implementing interventions aiming to improve early childhood development.

**WHAT IS ALREADY KNOWN ON THIS TOPIC**

**WHAT THIS STUDY ADDS**

- This study provides preliminary evidence from a multi-ethnic population study linking social support for women in pregnancy with their children’s communication and social-emotional development.
- There were some differences in the associations of maternal social support with child Strengths and Difficulties Questionnaire scores between migrant and non-migrant mothers, but associations were similar with Early Years Foundation Stage Profile outcomes.

**HOW THIS STUDY MIGHT AFFECT RESEARCH, PRACTICE OR POLICY**

- Interventions to improve social support in pregnancy could lead to better communication and social-emotional outcomes for children; it is likely to be helpful to consider different cultural contexts when designing such interventions.

## Introduction

 Social support, which refers to the existence and quality of social networks, is important in smoothing the transition from pregnancy to motherhood.^1^ Sources of support can include partners, family members, friends, community groups and healthcare professionals. Better and more social support networks are thought to buffer against stress during pregnancy and alleviate the challenges of pregnancy and motherhood.^2^,^4^ By contrast, absent or adverse social support networks can worsen maternal stress, mental health, loneliness and isolation with detrimental effects on child emotional and cognitive ability.^5 6^ While relationships between social support and mental health in pregnancy have been well studied, a smaller body of evidence suggests higher levels of social support during pregnancy may be associated with better child outcomes, including socio-emotional problems, cognitive ability, intelligence and child development.^27^,^14^

No study to date has explored whether these associations differ between migrant and non-migrant mothers. Migrant women face multiple compounding stressors arising from the intersection of gender, race, social class, immigration and language.^15^ Pregnant women who have migrated to an unfamiliar context away from the communal or collective culture of their birth country face multiple challenges during and after pregnancy, notably related to the loss of social support structures, and the challenge of constructing maternal identity across cultures.^416^,^19^ Migrant women are at higher risk of adverse pregnancy and perinatal health outcomes, and subsequently, worse child outcomes, with access to professional care and support obstructed by structural, organisational, social, personal and cultural barriers.^18 20 21^

In this study, we aimed to assess the association between social support and child development in the Born in Bradford (BiB) birth cohort from the North of England. We also explored whether maternal migrant status modified the associations.

## Methods

### Study setting and data source

Bradford is a city situated in West Yorkshire, England, with a population around 560 200.^22^ The city has high levels of deprivation, ranked as the 13th most deprived district in England.^23^ Around 80% of the Bradford city population are born in England and 8% are of Pakistani origin, while in Bradford district, around 26% have Pakistani heritage.^24 25^

BiB is a multi-ethnic birth cohort established in 2007, which recruited 12 453 women between March 2007 and November 2010, including 13 776 pregnancies resulting in 13 740 live births.^26^ Mothers completed in-depth surveys during pregnancy and consented to the linkage of routinely collected health and education data. A number of follow-up assessments of children have since been undertaken, including the Growing Up study.^26^,^28^

### Measures

#### Outcomes

First, from routinely collected education data linked to BiB records, we used the statutory Early Years Foundation Stage Profile (EYFSP) assessments (2012/2013), administered in English schools at the end of the first year of schooling (aged 4–5 years) to assess school readiness. Children were recorded as emerging, expected or exceeding in different learning areas, with an emerging classification indicating development goals had not yet been met. Using the Communication and Language assessments, we categorised listening and attention, understanding and speaking results into whether a participant had met the expected level or not for each area. We then derived a binary total communication variable as our outcome, assessing if a participant had not met targets in one or more areas of the Communication and Language assessment. We did the same for the Personal, Social and Emotional development assessments, which measured self-confidence and self-awareness, managing feelings and behaviour and making relationships. These outcomes were recoded as missing for participants classified by the school as ‘absent for long periods or recently arrived’ (n=4). There is no evidence for the validity of EYFSP across different socioeconomic or ethnic groups.

Second, we used the parent-reported Strengths and Difficulties Questionnaire (SDQ), a 25-item screening tool identifying emotional and behavioural problems, indicators of adverse child social-emotional development, from the Growing Up follow-up at age 7–11 years.^29^ It comprises five scales with four measuring difficulties (conduct problem, peer problems, emotional problems and hyperactivity), and one measuring positive prosocial behaviour. A total difficulties score, created by summing the four difficulties subscales (maximum score 40), was used as our primary outcome, with all five subscales (maximum score 10) used as secondary outcomes. Subscales were also summed to provide externalising (hyperactivity and conduct) and internalising (emotional and peer problem) scores, which were used as secondary outcomes. Additionally, we used a cut-off of 13 to dichotomise normal versus borderline/abnormal scores. Previous research in the UK has shown SDQ to be acceptable for use across different ethnicities.^30^

### Exposures

Maternal social support was assessed using a series of questions (online supplemental table 1) included in the baseline questionnaire during pregnancy, focusing on support from partners and family. Social support was not asked in all phases of baseline data collection and we included participants from the phase of data collection where questions were asked about both social support and mental health. Responses to questions were scored from 1 to 5 on a Likert scale or binary yes/no (1/0). We summed the seven items of social support, and maximum scores were 22, with higher scores representing higher levels of social support. We standardised the social support variable to have a mean of zero and an SD of 1 by subtracting the mean and dividing by the SD; this standardised variable was used in the analysis. We did not include participants if they had responded to less than four of the social support questions. If a participant responded to four or more questions, we prorated their score and included them in the analysis.

### Covariates

We used the 28-item General Health Questionnaire (GHQ-28) administered during pregnancy to assess maternal mental health (coded continuously) as a continuous variable. Ethnicity was categorised into White, Asian/Asian British, Black/Black British/Caribbean or African and other. A composite measure of socioeconomic position (SEP) was used, created by BiB researchers due to the complexity of measuring SEP in this multi-ethnic population, with the following categories: (1) least deprived and most educated, (2) employed but not materially deprived; (3) employed, no access to money, (4) benefits receipt but coping, (5) most deprived.^31^ Because of this, we do not include variables that make up the composite measure, for example, maternal education, as additional covariates. Maternal age (years) and child age (months) were used as continuous variables. Child sex was categorised as male or female. We also controlled for binary measures of maternal smoking and maternal drinking during pregnancy, both self-reported at baseline. A binary measure of migrant/non-migrant was derived. Migrant mothers were categorised as such if they were not born in England, Scotland, Wales or Northern Ireland.

### Data analysis

Using different subsets of the cohort for each outcome (one for SDQ, one for EYFSP) due to the different sample sizes available, we described participants’ characteristics using means and medians for continuous variables and counts and percentages for categorical variables. We compared the distribution of demographic variables in participants with complete and incomplete data using t-test and χ^2^ test. We also used Pearson’s correlation coefficient, analysis of variance (ANOVA) and χ^2^ test to explore the associations between social support, maternal migrant status and other sociodemographic variables. Using linear and logistic regression models, associations between social support and each outcome were estimated using an unadjusted model (model 1), an adjusted model (model 2—maternal age, composite SEP, mother migrant status, child sex, ethnicity, smoked during pregnancy and drank during pregnancy) and a fully adjusted model (model 2 plus maternal mental health). Maternal mental health was included as a covariate in the final model because it could be either a cause or consequence of social support, and therefore could either confound or mediate our association of interest. With the available data, it is not possible to separate these two concepts; therefore, model 3 must be interpreted with this limitation in mind. We then assessed whether mother’s migrant status modified the associations by stratifying our previous analyses. To estimate statistical evidence for interactions, we performed likelihood ratio tests to compare models with and without an interaction term between social support and mother’s migrant status, using the set of covariates in model 3. We used a complete case analysis approach because there were insufficient auxiliary variables in the available data to impute either social support or the outcomes of interest.

Coefficients were produced from the linear regression estimates and odds ratios (OR) were presented for outputs from logistic regression models. All analyses were conducted in STATA V.18.

## Results

**Figure 1:**
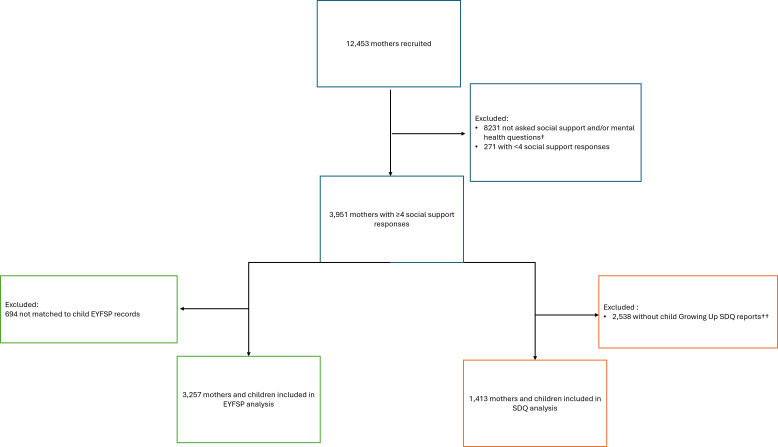
Study flow chart showing inclusion of Born in Bradford participants for two separate analyses. EYFSP, Early Years Foundation Stage Profile; SDQ, Strengths and Difficulties Questionnaire. †Maternal social support and mental health measures were not asked in all phases of baseline data collection to all mothers. ††Child does not have parent-reported Growing Up SDQ reports as mother may not have been asked or did not respond.

We analysed data from 3257 and 1413 participants with EYFSP and SDQ data, respectively. Figure 1 highlights how our final sample size was derived. Online supplemental table 1 describes the differences between included and excluded participants in both subsets of our data.

Descriptive statistics for each subset of the cohort are presented in table 1. There was an approximately equal split between male and female children; over half of the sample were of Asian ethnicity. Around 40% of mothers were migrants in the SDQ subsample, compared with approximately 30% in the EYFSP sample. In the SDQ sample, 57.5% of mothers were born in England and 31.6% were born in Pakistan, while in the EYFSP sample, these figures were 67.1% and 23.5%, respectively. In the EYFSP, 17.1% of children had not met at least one communication marker, and 15.9% had not met at least one personal, social and emotional development marker. Scores were skewed towards better scores in the SDQ overall score and subscales.

**Table 1:** Summary statistics of social support, developmental outcomes and covariates in each subsample of the Born in Bradford cohort

	SDQ cohort	EYFSP cohort
	N=1413*	N=3257*
	Mean (SD)	
Maternal age at baseline (years)	28.2 (5.5)	27.5 (5.6)
	Median (Q1–Q3)	
Maternal social support score (at baseline)	19 (16–21)	19 (16–21)
No. living in household	4 (3–5)	2 (2–5)
GHQ-28 score (at baseline)	21 (15–28)	21 (14–28)
SDQ (aged 7–11 years)		
Total SDQ score	8 (4–12)	–
Prosocial	9 (8–10)	–
Emotional	2 (0–3)	–
Conduct	1 (0–2)	–
Hyperactivity	3 (1–5)	–
Peer problems	1 (0–2)	–
Internalising	3 (1–5)	–
Externalising	5 (2–7)	–
	N (%)	
SDQ		
Normal	1127 (79.8)	–
Borderline/Abnormal	286 (20.2)	–
Communication and Language (EYFSP; aged 4–5 years)		
Not met in listening and attention	–	453 (13.9)
Not met in understanding	–	464 (14.3)
Not met in speaking	–	462 (14.2)
Not met at least one communication outcome	–	557 (17.1)
PSE (EYFSP; aged 4–5 years)		
Not met in self-confidence and self-awareness	–	393 (12.1)
Not met in managing feelings and behaviour	–	425 (13.1)
Not met in making relationships	–	353 (10.8)
Not met at least one PSE outcome	–	519 (15.9)
Male child	750 (53.1)	1655 (50.8)
Parental cohabitation status		
Married and living with partner	1054 (74.6)	2049 (62.9)
Not married and living with partner	184 (13.0)	609 (18.7)
Not living with partner	175 (12.4)	599 (18.4)
SEP		
Least deprived and most educated	307 (21.7)	603 (18.5)
Employed, not materially deprived	258 (18.3)	653 (20.1)
Employed, no access to money	241 (17.1)	557 (17.1)
Benefits but coping	418 (29.6)	910 (27.9)
Most deprived	189 (13.4)	534 (16.4)
Ethnicity		
White	490 (34.7)	1488 (45.7)
Asian/Asian British	865 (61.2)	1630 (50.1)
Black/Black British/Caribbean or African	18 (1.3)	42 (1.3)
Other	40 (2.8)	97 (3.0)
Smoked during pregnancy	166 (11.8)	587 (18.0)
Alcohol during pregnancy/3 months prior	305 (21.6)	903 (27.7)
Mother born outside UK (migrant)	595 (42.1)	1054 (32.4)

* Shown with no missing data for any variable, (complete case).

EYFSP, Early Years Foundation Stage Profile; GHQ-28, 28-item General Health Questionnaire; PSE, Personal, Social and Emotional Development; SDQ, Strengths and Difficulties Questionnaire; SEP, socioeconomic position.

Table 2 presents the associations between social support, maternal migrant status and other selected variables in the SDQ subset (EYFSP shown in online supplemental table 3), using Pearson’s correlation coefficient, ANOVA and χ^2^ test. Mean levels of social support were lower in mothers with lower educational levels, lower SEP, not employed, not living with partner and worse financial status. Higher levels of social support were associated with better maternal mental health and older mothers. There were minimal differences in mean social support between ethnicities or according to mother’s migrant status.

**Table 2:** Associations between social support, maternal migrant status and selected variables in Strengths and Difficulties Questionnaire dataset

N=1413	Mean social support	P value	Non-migrant mother	Migrant mother	P value
	Correlations (Pearson’s)				
Maternal age at baseline (years)	r=0.068	0.011	–	–	
No. living in household	r=0.005	0.845	–	–	
Maternal mental health	r=−0.309	<0.001	–	–	
			Mean (p values from ANOVA)		
Maternal age at baseline (years)	–	–	27.7	28.9	<0.001
Household size	–	–	3.8	5	<0.001
Maternal mental health	–	–	22.9	22	0.101
	Mean social support score (p values from ANOVA)		N (%) (p values from χ^2^ test)		
Maternal education		<0.001			<0.001
<5 GCSE	17.1		96 (11.7)	160 (26.9)	
5 GCSE	17.6		234 (28.6)	154 (25.9)	
A-level	18.6		208 (25.4)	50 (8.4)	
Higher than A-level	18.5		211 (25.8)	213 (35.8)	
Other	17.4		62 (7.6)	7 (1.2)	
Do not know/Foreign unknown	15.6		7 (0.86)	11 (1.9)	
Paternal education		<0.001			<0.001
<5 GCSE	17.3		97 (11.9)	81 (13.6)	
5 GCSE	18.1		227 (27.8)	116 (19.5)	
A-level	17.8		110 (13.5)	52 (8.7)	
Higher than A-level	18.6		180 (22.0)	211 (35.5)	
Other	17.6		59 (7.2)	10 (1.7)	
Do not know/Foreign unknown	17.4		145 (17.7)	125 (21.0)	
Mother employed		<0.001			<0.001
Yes	18.5		474 (58.0)	144 (24.2)	
No	17.5		344 (42.1)	451 (75.8)	
Socioeconomic position		<0.001			<0.001
Least deprived and most educated	19		169 (20.7)	138 (23.2)	
Employed not materially deprived	18.5		218 (26.7)	40 (6.7)	
Employed no access to money	17.7		139 (17.0)	102 (17.1)	
Benefits but coping	17.8		174 (21.3)	244 (41.0)	
Most deprived	16.3		118 (14.4)	71 (11.9)	
Ethnicity		0.972			<0.001
White	17.9		438 (53.6)	52 (8.7)	
Asian/Asian British	17.9		352 (43.0)	513 (86.2)	
Black/Black British/Caribbean or African	17.9		5 (0.6)	13 (2.2)	
Other	18.2		23 (2.8)	17 (2.9)	
Maternal migrant status		0.327	–	–	
Non-migrant	18				
Migrant	17.8				
Cohabitation status		<0.001			<0.001
Married and living with partner	18.2		498 (60.9)	556 (93.5)	
Not married and living with partner	17.6		165 (20.2)	19 (3.2)	
Not living with partner	16.5		155 (19.0)	20 (3.4)	
Managing financially		<0.001			<0.001
Living comfortably	18.8		230 (28.2)	143 (24.0)	
Doing alright	18.1		317 (38.8)	261 (43.9)	
Just about getting by	17.2		233 (28.5)	149 (25.0)	
Quite difficult	16.5		33 (40)	31 (5.2)	
Very difficult	15.8		4 (0.5)	11 (1.9)	

Pearson’s correlation coefficient was used to explore associations between two continuous variables; ANOVA was used where variables were continuous and categorical; χ2 test was used for two categorical variables. GCSE = General Certificate of Secondary Education

ANOVA, analysis of variance.

Similarly, there were associations between maternal migrant status and all variables we assessed, other than maternal mental health and social support. Compared with non-migrant mothers, based on the observed percentages, migrant mothers were older, had lower levels of employment, lower SEP, larger households and higher likelihood of being married and living with a partner.

After adjusting for all covariates, we found that better levels of social support were associated with lower odds of children missing the expected standard in EYFSP assessments of school-readiness (table 3). One SD higher social support score was associated with 13% lower odds for missing each communication target and ranged between 15% and 17% lower for personal, social and emotional development outcomes.

**Table 3:** Association between social support and child development outcomes (EYFSP (binary) and SDQ scores (continuous and binary)), from linear and logistic regression models

	Model 1	Model 2	Model 3
	EYFSP		
	OR per SD higher maternal social support score (95% CI)		
Communication and Language			
Not met listening and attention	0.82 (0.75, 0.90)	0.87 (0.79, 0.96)	0.87 (0.78, 0.97)
Not met understanding	0.83 (0.76, 0.91)	0.88 (0.80, 0.98)	0.87 (0.79, 0.97)
Not met speaking	0.85 (0.77, 0.93)	0.90 (0.81, 0.99)	0.87 (0.79, 0.97)
Not met communication and language (overall combined)	0.83 (0.76, 0.90)	0.88 (0.80, 0.96)	0.87 (0.79, 0.95)
Personal, Social and Emotional			
Not met self-confidence/self-awareness	0.81 (0.73, 0.89)	0.86 (0.77, 0.96)	0.85 (0.76, 0.95)
Not met managing feelings and behaviour	0.79 (0.72, 0.87)	0.84 (0.76, 0.93)	0.84 (0.75, 0.93)
Not met making relationships	0.82 (0.74, 0.91)	0.88 (0.79, 0.98)	0.88 (0.78, 0.99)
Not met personal, social, emotional (overall combined)	0.79 (0.72, 0.86)	0.84 (0.77, 0.93)	0.83 (0.75, 0.92)
	**SDQ**		
	**Mean difference per SD higher maternal social support score (95% CI)**		
SDQ			
Total SDQ score 40; 8 (4–12)	−1.20 (−1.52, –0.88)	−0.96 (−1.28, –0.65)	−0.65 (−0.98, –0.32)
Prosocial 10; 9 (8–10)	0.18 (0.09, 0.28)	0.17 (0.07, 0.28)	0.13 (0.02, 0.23)
Emotional 10; 2 (0–3)	−0.26 (−0.37, –0.14)	−0.20 (−0.32, –0.09)	−0.09 (−0.21, 0.03)
Conduct 10; 1 (0–2)	−0.32 (−0.41, –0.24)	−0.26 (−0.35, –0.18)	−0.23 (−0.32, –0.14)
Hyperactivity 10; 3 (1–5)	−0.33 (−0.46, –0.19)	−0.25 (−0.39, –0.11)	−0.15 (−0.29, 0.00)
Peer problems 10; 1 (0–2)	−0.30 (−0.39, –0.20)	−0.24 (−0.34, –0.15)	−0.18 (−0.28, –0.08)
Internalising 20; 3 (1–5)	−0.55 (−0.72, –0.38)	−0.45 (−0.62, –0.27)	−0.27 (−0.45, –0.09)
Externalising 20; 5 (2–7)	−0.65 (−0.85, –0.45)	−0.52 (−0.71, –0.32)	−0.38 (−0.58, –0.18)
	**OR per SD higher maternal social support score (95% CI)**		
Borderline/Abnormal	0.69 (0.61, 0.78)	0.72 (0.63, 0.82)	0.79 (0.69, 0.91)

Each SDQ subscale is accompanied by its total possible score, as well as the median (IQR).

Coefficients are beta-coefficients (SDQ) or ORs (SDQ/EYFSP outcomes) per SD higher social support score.

Model 1—unadjusted associations; model 2—adjusted for maternal age, a derived variable of socioeconomic position, mother’s country of birth, ethnicity, smoked during pregnancy, drinking during pregnancy; child’s sex and child’s age; model 3: model 2 plus maternal mental health measured by General Health Questionnaire score.

EYFSP, Early Years Foundation Stage Profile; SDQ, Strengths and Difficulties Questionnaire.

Higher levels of social support were associated with better scores (fewer problems) on all SDQ subscales in regression analyses (table 3). After adjustment for all covariates, there was strong evidence of associations between higher social support and lower overall SDQ, conduct, peer problems, internalising and externalising scores and between higher social support and higher prosocial scores. One SD higher social support was associated with 0.96 (95% CI −1.28 to –0.65) lower overall SDQ scores before adjustment for maternal mental health, and 0.65 (95% CI −0.98 to –0.32) lower scores after adjustment for all variables. We did not find strong evidence of associations between social support and the emotional subscale, with estimates attenuated towards the null after adjustment for maternal mental health (before adjustment: β=−0.20 (95% CI –0.32, –0.09); after adjustment: β=−0.09, 95% CI −0.21, 0.03).

The likelihood ratio test for interaction did not provide evidence that mother’s migrant status moderated the relationships between social support and EYFSP outcomes, although associations were often more precise in non-migrant mothers.

We found evidence that migrant status moderated the relationship between social support and overall SDQ scores, conduct subscale, hyperactivity subscale, peer problems and externalising scores (table 4). In all of the moderated outcomes, we found that associations between higher levels of social support and better social-emotional development (SDQ) were stronger in children of non-migrant mothers. For example, 1 SD higher social support in non-migrant mothers was associated with 0.34 lower conduct scores (95% CI −0.46 to –0.21), whereas the magnitude of the association was nearly five times smaller in migrant mothers (β=−0.07, 95% CI −0.20, 0.05, p value for interaction=0.003).

**Table 4:** Association between social support and child development outcomes (EYFSP outcomes (binary) and SDQ (continuous and binary)), stratified by mother’s migrant status from linear and logistic regression models

	Model 1		Model 2		Model 3		Test for interaction (p values) (model 3)
	Non-migrant mother	Migrant mother	Non-migrant mother	Migrant mother	Non-migrant mother	Migrant mother	
	EYFSP (OR per SD higher maternal social support score (95% CI))						
Communication and Language							
Not met listening and attention	0.81 (0.72, 0.91)	0.85 (0.72, 0.99)	0.86 (0.76, 0.98)	0.89 (0.75, 1.05)	0.87 (0.76, 0.99)	0.88 (0.73, 1.04)	0.852
Not met understanding	0.83 (0.74, 0.94)	0.84 (0.72, 0.98)	0.89 (0.78, 1.01)	0.89 (0.75, 1.04)	0.87 (0.76, 0.99)	0.88 (0.75, 1.04)	0.883
Not met speaking	0.82 (0.73, 0.93)	0.91 (0.78, 1.06)	0.87 (0.76, 0.99)	0.96 (0.82, 1.13)	0.83 (0.72, 0.95)	0.95 (0.80, 1.13)	0.363
Not met communication and language (overall combined)	0.81 (0.73, 0.90)	0.86 (0.75, 1.00)	0.86 (0.77, 0.97)	0.91 (0.78, 1.07)	0.85 (0.75, 0.96)	0.90 (0.77, 1.06)	0.639
Personal, Social and Emotional							
Not met self-confidence/self-awareness	0.80 (0.70, 0.90)	0.84 (0.71, 0.99)	0.86 (0.75, 0.98)	0.87 (0.73, 1.03)	0.84 (0.73, 0.97)	0.86 (0.71, 1.03)	0.849
Not met managing feelings and behaviour	0.74 (0.66, 0.83)	0.90 (0.76, 1.07)	0.80 (0.71, 0.90)	0.94 (0.79, 1.13)	0.78 (0.68, 0.89)	0.95 (0.79, 1.15)	0.095
Not met making relationships	0.78 (0.69, 0.89)	0.92 (0.76, 1.11)	0.85 (0.74, 0.97)	0.94 (0.77, 1.15)	0.84 (0.73, 0.97)	0.95 (0.78, 1.17)	0.229
Not met personal, social, emotional (overall combined)	0.76 (0.69, 0.85)	0.85 (0.73, 0.99)	0.83 (0.74, 0.93)	0.88 (0.75, 1.04)	0.81 (0.72, 0.92)	0.88 (0.74, 1.04)	0.364
	SDQ (mean difference per SD higher maternal social support score (95% CI))						
SDQ							
Total SDQ score 40; 8 (4–12)	−1.60 (−2.03, –1.16)	−0.59 (−1.03, –0.16)	−1.21 (−1.65, –0.77)	−0.52 (−0.96, –0.08)	−0.89 (−1.35, –0.43)	−0.25 (−0.69, 0.19)	0.019
Prosocial 10; 9 (8–10)	0.24 (0.12, 0.37)	0.09 (−0.06, 0.25)	0.21 (0.08, 0.33)	0.11 (−0.05, 0.26)	0.17 (0.04, 0.31)	0.06 (−0.10, 0.21)	0.186
Emotional 10; 2 (0–3)	−0.29 (−0.44, –0.13)	−0.21 (−0.38, –0.05)	−0.21 (−0.37, –0.05)	−0.19 (−0.35, –0.03)	−0.07 (−0.24, 0.09)	−0.10 (−0.27, 0.06)	0.996
Conduct 10; 1 (0–2)	−0.44 (−0.56, –0.33)	−0.14 (−0.26, –0.02)	−0.35 (−0.47, –0.23)	−0.12 (−0.24, 0.01)	−0.34 (−0.46, –0.21)	−0.07 (−0.20, 0.05)	0.003
Hyperactivity 10; 3 (1–5)	−0.47 (−0.65, –0.29)	−0.10 (−0.32, 0.11)	−0.37 (−0.55, –0.18)	−0.08 (−0.29, 0.14)	−0.26 (−0.45, –0.06)	0.02 (−0.20, 0.23)	0.04
Peer problems 10; 1 (0–2)	−0.40 (−0.52, –0.28)	−0.14 (−0.28, 0.00)	−0.28 (−0.41, –0.16)	−0.14 (−0.28, 0.00)	−0.22 (−0.35, –0.09)	−0.09 (−0.22, 0.06)	0.04
Internalising 20; 3 (1–5)	−0.69 (−0.92, –0.45)	−0.35 (−0.59, –0.11)	−0.49 (−0.74, –0.25)	−0.33 (−0.57, –0.09)	−0.30 (−0.55, –0.04)	−0.19 (−0.43, 0.05)	0.264
Externalising 20; 5 (2–7)	−0.91 (−1.17, –0.64)	−0.24 (−0.53, 0.05)	−0.72 (−0.98, –0.45)	−0.19 (−0.48, 0.09)	−0.59 (−0.87, –0.31)	−0.06 (−0.35, 0.23)	0.005
	OR per SD higher maternal social support score (95% CI)						
Borderline/Abnormal	0.64 (0.55, 0.74)	0.79 (0.62, 1.00)	0.69 (0.59, 0.81)	0.79 (0.62, 1.01)	0.76 (0.64, 0.89)	0.86 (0.67, 1.11)	0.235

For each SDQ subscale, the total possible score and median (IQR) are shown.

Coefficients are beta-coefficients (SDQ) or ORs (SDQ/EYFSP outcomes) per SD higher social support score.

Model 1—unadjusted estimates; model 2—adjusted for maternal age, a derived variable of socioeconomic position, child’s sex, ethnicity, smoked during pregnancy; drinking during pregnancy; child’s age; model 3—model 2 plus maternal mental health measured by General Health Questionnaire score.

EYFSP, Early Years Foundation Stage Profile; SDQ, Strengths and Difficulties Questionnaire.

## Discussion

In this analysis of a multi-ethnic birth cohort, we found that higher levels of social support during pregnancy were associated with greater odds of their children meeting the expected standard in EYFSP assessments of school readiness (age 4–5 years), and scoring better for social-emotional development, assessed by SDQ (age 7–11 years), after adjustment for sociodemographic factors and measures of maternal mental health.

Our findings are similar to what has been found in the limited existing literature, which found higher levels of perinatal social support to be associated with better infant social-emotional development, although with small effect sizes.^9^ In a similar age group to this study (aged 8 years), one study explored cognitive ability and also found higher levels of social support were associated with improved cognitive outcomes.^10^

Social support in pregnancy deserves greater attention as a measure with multifaceted potential to link interventions and outcomes. Our findings underscore the need for mechanism-oriented studies considering antenatal protective factors for child development.^10^ While mental health services are routinely offered in the UK during pregnancy, fewer population-wide avenues to improving social support are available. Research on interventions to improve social support is limited, particularly in the UK, but ranges from psychoeducation to additional midwife support to community befrienders, with the latter having particular success in asylum-seekers/refugees.^32^,^34^ Shared language, values and group identity-building may have particular importance, which may be significant in thinking about social support as experienced by migrants affected by many marginalising processes that impact well-being and social exclusion.^4 35^

In this study, social support levels were more strongly associated with SDQ scores in children with non-migrant mothers than migrant mothers. Associations between maternal social support and EYFSP outcomes did not show equivalent differences by maternal migration status. Distributions of mothers’ scores for social support did not differ by maternal migrant status. Recent evidence highlighted differences in child EYFSP educational outcomes between children of first-generation and second-generation migrants within BiB.^36^ The simple stratification of migration into ‘born in the UK or not’ clearly does not enable us to explore the nuances of intergenerational experiences of migration and settlement, social support and/or raising children, and further research is needed to explore this.

It is also essential to interpret the findings within the study context. BiB is a unique cohort that has rich ethnic diversity with large minority ethnic communities and therefore, the lack of difference in prevalence of social support for non-migrant and migrant women may not be representative of other areas. In Bradford, there is a large, third-generation settled Pakistani community, meaning that while women who have recently moved from Pakistan may not have extensive social support networks, they may still benefit from living in an environment that offers cultural and religious familiarity and support.^37^ Research in Bradford has found that the social cohesion of this environment may have been protective for maternal mental health during the pandemic.^38^ Similarly, highly ethnically diverse neighbourhoods may offer protective effects for migrant children.^39 40^ Our findings could indicate different typical routes to positive social support across levels of affluence and education, by ethnicity and/or intergenerationally for more recent and more established migrant families. Further research is needed to explore these issues in detail.

### Strengths and limitations

Our study used a multi-ethnic birth cohort, with high levels of participants from South Asia, allowing a large sample for each stratified group. It represents the first attempt to explore the modifying effects of maternal migrant status on the associations between social support and child development.

However, our measure of social support has not been validated or previously been used as a composite measure, and it may not express the range of possible cultural differences in social support across the population studied, for example, if it did not address extra-familial support (eg, from community or health professionals), or sufficiency of support. Social support is not a unitary construct, nor universally beneficial, and the questions asked in this study may have had different relevance for women in different contexts. Additionally, we only had data for social support in pregnancy and could not examine subsequent maternal social support, and therefore, we do not account for social support levels postbaseline. In addition to social support, other measures included in our study may not be valid across different cultures, languages and experiences of migration and results should be interpreted in the context of this.

Our migration stratification did not take into account how long the mother had been in England, their specific country of birth or their family circumstances. The selection of our subsample could be biased as we used a complete case analysis, despite missing data, due to a lack of auxiliary variables to impute missing data. Our approach may have led to an under-representation of some groups, for example, migrant mothers (online supplemental table 2), and could lead to an overestimation of support given, particularly if those excluded were less familiar with English or had spent less time in the country. However, their inclusion may strengthen the association between social support and the chosen outcomes. Finally, residual confounding between maternal social support and child outcomes is possible as we did not control for some variables, such as premature birth, interpersonal violence or disability.

## Conclusion

Our study within the BiB cohort found associations between maternal social support during pregnancy, school-assessed outcomes (EYFSP) at age 4–5 years, and parent-assessed social-emotional development (SDQ) at age 7–11 years. Further research is needed on whether and how interventions that could improve social support in pregnancy have the potential to improve outcomes for children; it is likely to be useful to consider different cultural contexts when designing such interventions. Overall, our findings underline the importance of social support in pregnancy for children’s outcomes for both UK-born and migrant families, and the need for better understanding of mechanisms and interventions.

## Supplementary material

10.1136/archdischild-2025-328885 online supplemental file 1

## Data Availability

Data may be obtained from a third party and are not publicly available.
