# Supplementary material for: Maternal social support and child developmental outcomes: an analysis of the Born in Bradford cohort
Source: Arch Dis Child. 2025 Jun 24;110(12):e328885. doi: 10.1136/archdischild-2025-328885 (PMC12703351; doi:10.1136/archdischild-2025-328885)
Supplement: online supplemental file 1 [file archdischild-110-12-s001.docx]

Supplementary Materials

Table 1. Questions used to derive the social support variable at baseline

| Social Support | Response categories |
| --- | --- |
| 1. My husband/partner doesn‘t seem to listen to me 2. I wish there was more warmth and affection between us 3. I feel closely attached to my family 4. My family takes notice of my opinions 5. Sometimes I feel excluded in my own family | Totally agree  Agree  Neither agree nor disagree  Disagree  Totally disagree |
| 1. Do you eat at least one meal at home with your husband, partner, family or friends? 2. Do you have people living with you –relatives or friends- that you wish weren‘t there? | Yes  No  Prefer not to say |
| Max score: 22 | |

Table S2. Description of demographic measures in participants with complete data compared with participants with missing data or lost to follow-up

|  | SDQ | | | EYFSP | | |
| --- | --- | --- | --- | --- | --- | --- |
|  | Included  n = 1,413 | Excluded  n = 12, 082 | p-value | Included  n = 3,257 | Excluded  n = 10,475 | p-value |
|  | Means | | | | | |
| Maternal age | 28.2 | 27.2 | <0.001 | 27.51 | 27.2 | 0.019 |
| No. living in household | 4.33 | 4.08 | <0.001 | 4.05 | 4.14 | 0.071 |
| Maternal mental health | 22.5 | 23.3 | 0.011 | 22.4 | 23.6 | <0.001 |
|  | % | | | | | |
| Maternal education  <5 GCSE  5 GCSE  A-level  Higher than A-level  Other  Don’t know/ Foreign Unknown | 18.12  27.46  18.26  30.01  4.88  1.27 | 22.1  31.3  13.9  25.0  5.6  2.3 | <0.001 | 19.6  30.6  18.3  25.4  4.6  1.5 | 22.4  30.7  12.9  25.7  5.9  2.4 | <0.001 |
| Paternal education  <5 GCSE  5 GCSE  A-level  Higher than A-level  Other  Don’t know/ Foreign Unknown | 12.6  24.3  11.5  27.7  4.9  19.1 | 15.8  24.0  10.1  24.8  4.5  20.9 | 0.005 | 14.5  25.3  10.6  23.8  5.0  20.9 | 15.7  23.5  10.2  25.7  4.3  20.6 | 0.047 |
| Mother not employed | 56.3 | 55.9 | 0.080 | 54.2 | 56.7 | 0.011 |
| Socioeconomic position  Least deprived and most educated  Employed not materially deprived  Employed no access to money  Benefits but coping  Most deprived | 21.7  18.3  17.1  29.6  13.4 | 19.4  20.1  14.9  29.3  16.3 | 0.003 | 18.5  20.1  17.1  27.9  16.4 | 20.2  19.8  14.4  29.9  15.7 | 0.001 |
| Ethnicity  White  Asian/Asian British  Black/Black British/Caribbean or African Other | 34.7  61.2  1.3  2.8 | 43.1  51.3  2.2  3.4 | <0.001 | 45.7  50.1  1.3  3.0 | 41.1  53.1  2.4  3.5 | <0.001 |
| Mother not UK born (migrant) | 42.1 | 48.4 | <0.001 | 32.4 | 52.3 | <0.001 |
| Household size | 4.33 | 4.08 | <0.001 | 4.05 | 4.14 | 0.071 |
| Cohabitation status  Married and living with partner  Not married and living with partner  Not living with partner | 74.6  13.0  12.4 | 64.4  18.6  17.0 | <0.001 | 62.9  18.7  18.4 | 66.9  17.4  15.7 | <0.001 |
| Managing financially  Living comfortably  Doing alright  Just about getting by  Quite difficult  Very difficult | 26.4  40.9  27.1  4.5  1.1 | 26.6  41.8  23.7  5.9  2.0 | 0.003 | 24.2  41.0  27.8  5.5  1.6 | 27.7  41.8  22.6  5.9  2.0 | <0.001 |
| SDQ = Strengths and Difficulties Questionnaire. EYFSP = Early Years Foundation Stage Profile.  Numbers of participants excluded differs between variables.  T-tests were used to assess the differences between being complete and continuous variables, χ^2^ was used for categorical variables. | | | | | | |

Table S3. Associations between social support, maternal country of birth and selected variables in Early Years Foundation Stage Profile (EYFSP) dataset

| n=3,257 | Mean Social Support | p-value | Mother born in UK | Mother not born in UK | p-value |
| --- | --- | --- | --- | --- | --- |
|  | *Correlations (Pearson’s)* | |  |  |  |
| Maternal age | r = 0.092 | <0.001 | - | - | - |
| Household size | r = 0.0003 | 0.986 | - | - | - |
| Maternal mental health | r = -0.299 | <0.001 | - | - | - |
|  |  |  | *Mean (p values from ANOVA)* | | |
| Maternal age | - | - | 27.0 | 28.7 | <0.001 |
| Household size | - | - | 3.63 | 4.93 | <0.001 |
| Maternal mental health | - | - | 22.7 | 21.8 | 0.023 |
|  | *Mean social support score (p values from ANOVA)* | | *n (%) ( χ^2^)* | | |
| Maternal education  <5 GCSE  5 GCSE  A-level  Higher than A-level  Other  Don’t know/ Foreign Unknown | 17.1  17.7  18.2  18.5  17.9  16.5 | <0.001 | 363 (16.5)  730 (33.1)  507 (23.0)  443 (20.1)  136 (6.2)  24 (1.1) | 276 (26.2)  267 (25.3)  88 (8.4)  384 (36.4)  14 (1.3)  25 (2.4) | <0.001 |
| Paternal education  <5 GCSE  5 GCSE  A-level  Higher than A-level  Other  Don’t know/ Foreign Unknown | 17.3  17.9  18.0  18.6  17.9  17.3 | <0.001 | 323 (14.7)  610 (27.7)  260 (11.8)  408 (18.5)  139 (6.3)  463 (21.0) | 149 (14.1)  213 (20.2)  84 (8.0)  367 (34.8)  23 (2.2)  218 (20.7) | <0.001 |
| Mother employed  Yes  No | 18.5  17.4 | <0.001 | 1228 (55.7)  975 (44.3) | 268 (25.4)  786 (74.6) | <0.001 |
| SEP  Least deprived and most educated  Employed not materially deprived  Employed no access to money  Benefits but coping  Most deprived | 19.1  18.5  17.4  17.7  16.4 | <0.001 | 364 (16.5)  570 (25.9)  372 (16.9)  504 (22.9)  393 (17.8) | 239 (22.7)  83 (7.9)  185 (17.6)  406 (38.5)  141 (13.4) | <0.001 |
| Ethnicity  White  Asian/Asian British  Black/Black British/Caribbean or African Other | 17.9  17.8  18.0  18.0 | 0.961 | 1384 (62.8)  743 (33.7)  10 (0.5)  66 (3.0) | 104 (9.9)  887 (84.2)  32 (3.0)  31 (2.9) | <0.001 |
| Maternal migrant status  Non-migrant  Migrant | 17.9  17.7 | 0.067 | - | - | - |
| Cohabitation status  Married and living with partner  Not married and living with partner  Not living with partner | 18.2  17.7  16.9 | <0.001 | 1080 (49.0)  573 (26.0)  550 (25.0) | 969 (91.9)  36 (3.4)  49 (4.7) | <0.001 |
| Managing financially  Living comfortably  Doing alright  Just about getting by  Quite difficult  Very difficult | 18.9  18.0  17.1  16.5  16.0 | <0.001 | 549 (25.0)  871 (40.0)  636 (28.9)  113 (5.1)  31 (1.4) | 237 (22.6)  463 (44.1)  266 (25.3)  65 (6.2)  20 (1.9) | 0.026 |
| Pearson’s correlation coefficient was used to explore associations between two continuous variables; ANOVA was used where variables were continuous and categorical and chi-squared was used for two categorical variables | | | | | |
